# Glacier thickness and ice volume of the Northern Andes

**DOI:** 10.1038/s41597-022-01446-8

**Published:** 2022-06-15

**Authors:** Maximillian Van Wyk de Vries, David Carchipulla-Morales, Andrew D. Wickert, Verónica G. Minaya

**Affiliations:** 1 University of Minnesota, Department of Earth & Environmental Sciences, Minneapolis, MN 55455 USA; 2 University of Minnesota, Saint Anthony Falls Laboratory, Minneapolis, MN 55414 USA; 3 School of Environmental Sciences, University of Liverpool, Liverpool, L3 5DA UK; 4 Escuela Politécnica Nacional, Departamento de Ingeniería Civil y Ambiental, Quito, 170525 Ecuador; 5 GFZ German Research Centre for Geosciences, Telegrafenberg, 14473 Potsdam, Germany

**Keywords:** Hydrology, Natural hazards

## Abstract

Tropical glacier melt provides valuable water to surrounding communities, but climate change is projected to cause the demise of many of these glaciers within the coming century. Understanding the future of tropical glaciers requires a detailed record of their thicknesses and volumes, which is currently lacking in the Northern Andes. We calculate present-day (2015–2021) ice-thicknesses for all glaciers in Colombia and Ecuador using six different methods, and combine these into multi-model ensemble mean ice thickness and volume maps. We compare our results against available field-based measurements, and show that current ice volumes in Ecuador and Colombia are 2.49 ± 0.25 km^3^ and 1.68 ± 0.24 km^3^ respectively. We detected no motion on any remaining ice in Venezuela. The overall ice volume in the region, 4.17 ± 0.35 km^3^, is half of the previous best estimate of 8.11 km^3^. These data can be used to better evaluate the status and distribution of water resources, as input for models of future glacier change, and to assess regional geohazards associated with ice-clad volcanoes.

## Abstract (summary)

**Table:** 

Measurement(s)	Glacier Thickness
Technology Type(s)	Velocity based inversion
Factor Type(s)	Ice thickness
Sample Characteristic - Environment	glacier
Sample Characteristic - Location	Northern Andes

## Background & Summary

The Northern Andes is a high-altitude mountain range in the inner tropics of Colombia, Ecuador, and Venezuela. Despite their proximity to the equator, many peaks in this region are glacierized^1^. These tropical glaciers represent both a valuable water resource to millions of people^2–7^ and a major source of geohazards. By storing water on multi-annual timescales, glaciers alleviate water shortages during dry seasons and in times of drought^8–12^. From a geohazards perspective, many of the Northern Andean glaciers are located on active volcanoes^13^. Eruptions from glaciated volcanoes commonly trigger damaging jokulhlaups (floods) and lahars (mudlflows)^13–16^. The most deadly volcanic disaster of the past century occurred in the Northern Andes, associated with volcano–ice interactions during the 1985 eruption of Nevado del Ruiz, Colombia^17,18^. Finally, many tropical glaciers also hold cultural significance^4,19^, for instance those on Chimborazo^20^.

Climate change has driven global glacier recession over the past decades^21^. This recession is particularly pronounced in tropical regions^21,22^, where glaciers may melt year-round due to the lack of temperature seasonality^23^. Many glaciers in the Northern Andes are forecast to disappear entirely over the course of the 21st century^24,25^. Better assessing the timing of this ice loss and forecasting its impact requires knowledge of the existing ice thickness and volume in the Northern Andes. To date, regional ice-volume assessments have focused on glacier-area change^26,27^.

In this study we combine remote sensing of glacier-surface velocities together with multiple ice-physics-based inversions to build a new database of present-day (2015–2021) ice thicknesses and volumes for all glaciers in Colombia, Ecuador, and Venezuela^28–37^. We validate our dataset against available field measurements of ice thickness and volume from the Northern Andes^38–42^, and compare these to previous global assessments^35,37^. For each glacier in the Northern Andes, our database includes an updated glacier-extent polygon, a 50-m-resolution gridded map of glacier-surface velocities, six different 50-m-resolution maps of ice thickness calculated using different methods, and a 50-m-resolution multi-model ensemble mean ice thickness map^43^. Each ice-thickness map has a complementary grid of ice-thickness uncertainty.

Our ice-thickness maps^43^ allow for an estimation of the current ice volume in Colombia, Ecuador, and Venezuela, and provide a baseline for future monitoring of volumetric ice loss. This database can support a range of future research objectives, including forecasts of glacier-mass loss and the timing of peak glacial runoff, assessments of water availability for major cities (e.g. Quito^5,44^), and improvements to regional volcanic hazard maps^14,15,18,40^.

## Methods

**Fig. 1:**
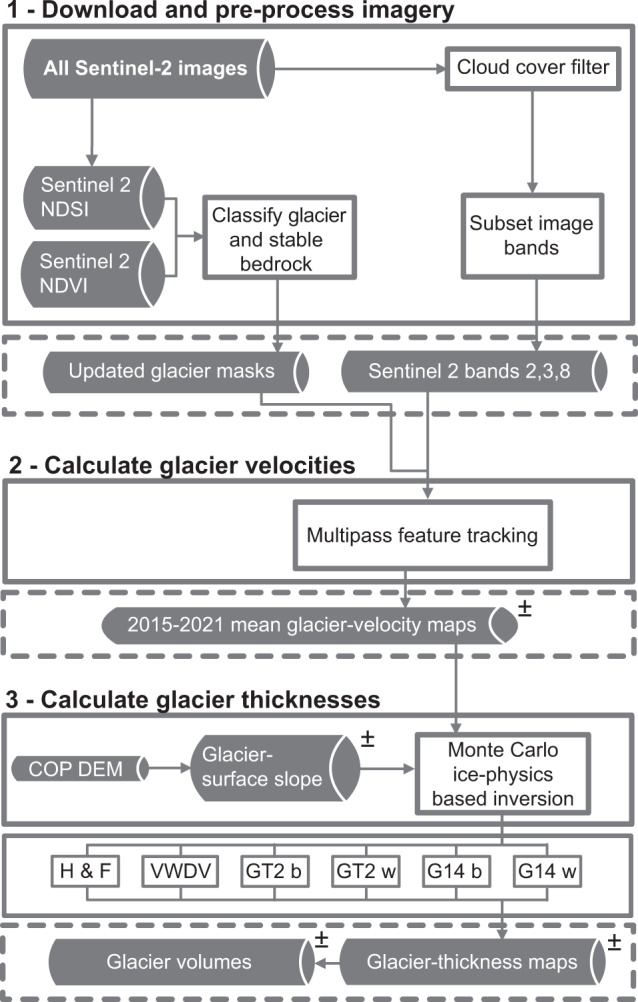
Steps involved in creating ice-thickness maps. Shaded boxes represent datasets, white boxes represent processes, and the ± symbol indicates that a dataset includes a measure of uncertainty. COP DEM = Copernicus Digital Elevation Model^47^; H F = mass-conservation-based approach^29,30^; VWDV = fully distributed velocity-based inversion from this study; GT2 b = basal-shear-stress-based basin-divided approach^31,34^; GT2 w = basal-shear-stress-based whole glacier approach^31,34^; G14 b = Gantayat *et al*.^32^ basin-divided approach^32^; G14 w = Gantayat *et al*.^32^ whole glacier approach^32^.

We design a workflow for calculating ice thicknesses and ice volumes in multiple ways using glacier-surface topography, glacier-surface flow speed, glacier geometry, and glacier basal shear stress (Fig. 1).

### Step 1a. Satellite image download

We use Google Earth Engine^45^ to identify, pre-process, and download Sentinel-2 satellite imagery^46^ of all ice-covered areas in Colombia, Ecuador, and Venezuela. We (i) use Randolph Glacier Inventory (RGI^1^) polygons to identify the glaciers, (ii) manually delete polygons for from areas that show exposed bedrock for at least part of the year, and therefore have been transformed from glaciers to seasonal snowfields, (iii) buffer polygons with a 1 km margin, and (iv) merge overlapping polygons. This results in 6 extant glaciers in Ecuador (El Altar, Chimborazo, Illiniza Sur, Cotopaxi, Antisana, and Cayambe), 6 glaciers or glacier zones in Colombia (Nevado del Huila, Nevado de Tolima, Santa Isabel, Nevado del Ruiz, El Cocuy, and Sierra Nevada de Santa Marta), and one single glacier in Venezuela (La Corona, Pico Humboldt).

We clip every available Sentinel-2 image (from 2015 to 2021) to the area of interest from each ice-cover polygon. We include only images with less than 60% cloud cover based on the cloud mask from the Sentinel-2 quality assurance (QA) band. We average three bands (Green:B03, Red:B04, and Near infrared:B08) for use in feature tracking^36^. In addition to the three Sentinel-2 bands, we download the 1-arcsecond-resolution Copernicus DEM^47^ to provide ice surface slope for the ice-thickness inversion.

### Step 1b. Ice masking

We find that RGI polygons overestimate the spatial extent of the majority of glaciers in the Northern Andes, likely due to rapid glacier recession and persistent high-altitude snow cover throughout the year^1^. Any ice-volume calculation method using the RGI polygons^30,35,37^ is therefore likely to overestimate the true volume. Hence, we use a new ice-masking approach, which uses percentile analysis of satellite image timeseries to differentiate permanently glaciated regions from temporary snow cover. This script generates an ice index (using Sentinel-2 visible bands 2, 3, and 4, and shortwave infra-red bands 11 and 12) and a water index (using Sentinel-2 visible bands 2 and 3, and near infra-red band 8), and classifies pixels as ice or water when the respective index is below an optically calibrated threshold in 90% of individual images. This threshold filters out temporary snow cover but retains zones of persistent ice cover. Water pixels are merged with non-ice pixels and masked out, leaving a binary ice mask. Removing water pixels is important as these may otherwise be misclassified as ice: two recent global compilations mis-identify the El Altar crater lake as ice, and include it in their glacier volume calculations^35,37^. Due to its very high tephra cover, we manually delineate the Nevado del Ruiz glacier zone by hand using 3-m-resolution Planet DoveSat images collected on 10 and 12 February, 2020. We manually evaluate each glacier mask against low snow cover Sentinel-2 images from 2020 or 2021.

### Step 2. Velocity map generation

We use the feature-tracking toolbox GIV to calculate 50-m-resolution glacier-surface-velocity maps for each location, using a frequency-domain multi-pass image correlator^36^. We also calculate apparent displacements over the surrounding bedrock to correct for georeferencing errors and evaluate local noise levels. We include all image pairs with a minimum temporal separation of 3 months and a maximum temporal separation of 4 years. This allows us to compile a large dataset of image pairs at each glacier, ranging from 230 pairs at Nevado del Huila to 14,198 image pairs at Sierra Nevada de Santa Marta. Calculating a large number of individual velocity maps is advantageous, as the precision of mean velocity maps improves with the number of individual velocity maps stacked^36,48^. We do not consider glacier surges, as they have not been documented in this region and the local glacier characteristics are unfavorable to their occurrence^49^.

For each Sentinel-2 image pair, we calculate displacements using iteratively reducing multipass template matching following the standard GIV workflow. We use the standard GIV reference window sizes of 400 m, 200 m, and 100 m and a 50% window overlap, for a final velocity-map resolution of 50 m. Displacements are evaluated to sub-pixel precision in the final pass using a Gaussian sub-pixel estimator^36^. We convert each pair-wise displacement map into a velocity map by dividing it by the temporal separation between images. We filter each velocity map by removing the value for pixels which meet any of the following criteria: the velocity exceeds the maximum velocity threshold of 100 m.a^−1^, the signal to noise ratio is lower than 5, or the peak ratio is less than 1.3^36^. These thresholds were manually selected based on local tests to exclude the majority of pixels with erroneous velocity estimates based on comparison with neighboring pixels and external datasets^37^. We also exclude values that differ by more than 50% from their immediate neighbours (four surrounding cells) and 200% from the mean of their larger local area (25 surrounding cells), and interpolate across these now-empty pixels using the values of the remaining (i.e. valid) ice-speed pixels. We do not filter based on flow direction because most of the region’s glaciers flow radially outwards from mountain peaks. To correct for possible georeferencing errors, we subtract the median velocity over non-glacierized (stable) areas from the glacier velocity in the x and y directions for each image pair. We calculate a timeseries of velocities for each pixel, and after constructing this timeseries exclude pixels having a glacier speed in excess of 1.5 standard deviations from the mean^36^. Finally, we average all individual processed velocity maps into a mean velocity map covering the entire period. We crop mean velocity maps to the updated ice mask prior to inverting for ice thickness to exclude background noise over non-glacierized terrain from ice-volume calculations.

### Step 3. Ice-thickness calculation

Previous intercomparisons of glacier-thickness calculation methods have shown that, while no single approach is clearly superior to all others, the average of multiple different methodologies is generally more accurate than any single method^33,50^. We therefore use an ensemble of six different methods to calculate the thickness of all glaciers in our study area. Three of these methods use glacier surface flow speeds to invert for ice thickness^32,33,36^, two of these use a basal-shear-stress-based approach^28,31,33–35^, and one method uses a mass-conservation-based approach^29,30,35^.

#### 3a. Ice-velocity-based approaches

Glacier motion occurs through a combination of internal deformation, basal sliding, and subglacial sediment deformation. Ice-surface velocities *u*(*H*) may be written as a combination of internal deformation (*u*_*d*_) and basal velocity (*u*_*b*_; the sum of basal sliding and subglacial sediment deformation):

1 $$u(H)={u}_{d}(H)+{u}_{b}.$$

Here, the ice-thickness *H* denotes velocities (full and from internal deformation alone) that are evaluated at the ice surface.

We simplify this ice-flow equation based on the characteristics of the glaciers in this study area. Field studies have not revealed extensive subglacial sediment layers, thus subglacial sediment-deformation term should be at or near zero^23,41^. Glacial sliding requires warm-based ice and can be enhanced by water pressure^51^. In the tropical Northern Andes, seasonal temperature variations are low and the majority of glacier area is located in areas with a mean annual temperature below freezing^23^. As a consequence, basal sliding *u*_*b*_ likely accounts for a small proportion of the total glacier surface velocity. We therefore account for basal velocity *u*_*b*_ through a correction factor, *β*^34^, which corresponds to the fraction of glacier motion derived from basal sliding.

As a result, we directly relate internal deformation to glacier-surface velocity, and use this to compute a closed-form relationship between (unknown) ice thickness and observed ice velocity. Ice flows under its own weight, and the rate of internal deformation is a function of the ice thickness:^32,52,53^

2 $$(1-\beta )u(H)={u}_{d}(H)=\frac{2{A}_{c}}{n+1}{\tau }_{b}^{n}H.$$

Here, *τ*_*b*_ is the basal shear stress, *A*_*c*_ is the Arrhenius creep constant, and *n* is Glen’s flow exponent. Our use of the basal shear stress instead of the full driving stress for glacier motion comes from the shallow-ice approximation. Through this, we assume that local stresses induced by the ice are much greater than stresses induced by lateral coupling between columns of ice. Thin ice and steep slopes are characteristic of many Andean glaciers^38–41^. Both of these enhance the dominance of the basal shear stress within the full glacier driving stress, thereby supporting our use of the shallow-ice approximation.

We expand basal shear stress, *τ*_*b*_, into measurable parameters:

3 $${\tau }_{b}=f{\rho }_{i}gH{\rm{\sin }}(\alpha ).$$

Here, *f* is a shape factor accounting for lateral drag along the glacier margins^31,32,54^, *ρ*_*i*_ is the ice density, *g* is gravitational acceleration, *α* is the ice-surface slope angle (averaged over a length scale long enough that longitudinal coupling along the glacier flowline becomes negligible), and *H* is ice-thickness. We calculate the Arrhenius creep constant based on temperature:

4 $${A}_{c}={A}_{c}^{* }\exp \left(\frac{{Q}_{c}}{R}\left[\frac{1}{T}-\frac{1}{{T}^{* }}\right]\right),$$

with the constants being ${A}_{c}^{* }=2.4\cdot 1{0}^{-24}$, *Q*_*c*_ = 115 kJ mol^−1^, *R* ≈ 0.0083145 (the ideal gas constant), and T^*^ = 273 K^53^. We combine Eqs. 2, 3 and 4 and rearrange them to solve for ice-thickness:

5 $$H={\left(\frac{n+1}{2{\left(f{\rho }_{i}g\right)}^{n}{A}_{c}^{* }\exp \left(\frac{{Q}_{c}}{R}\left[\frac{1}{T}-\frac{1}{{T}^{* }}\right]\right)}\right)}^{1/(n+1)}{\left(\frac{u(H)(1-\beta )}{{\rm{\sin }}{(\alpha )}^{n}}\right)}^{1/(n+1)}$$

Here, the first term contains constants and parameters and the second term contains observations obtained from GIV (*u*_*H*_) and a digital elevation model (sin(*α*)).Thus, the only unknown required to solve for ice-thickness, *H*, is ice-surface velocity.

We implement this equation in three different ways, one novel to this study and two based on the work of Gantayat *et al*.^32^. Our implementation solves Eq. 5 at each location using the full two-dimensional ice surface flow speed and topographic slope fields (VWDV model). Gantayat *et al*.’s approach^32^ divides the glacier into 100-m elevation bands and computes mean ice-surface flow speed and topographic slope for each elevation band, which we implement in two different ways. First, we calculate elevation-band-averaged flow speed and slope for whole ice caps, defined as isolated clusters of ice-masked pixels calculated in Step 1b (G14 w model). This approach is closer to the original method^32^ but may average across multiple outlet glaciers from a single ice cap. Therefore, we also use TopoToolbox^55^ to divide ice caps into individual ice-drainage basins based on topographic slope. We then calculate elevation-band-averaged flow speed and slope for each elevation band within each individual ice basin (G14 b model). This approach can better honor the variable dynamics of outlet glaciers from ice caps, but may result in artificial step changes in ice thickness at the boundaries between basins.

The representative length scale over which glacier surface slope is physically significant (longitudinal coupling length) is a function of the local ice thickness. When implementing our fully distributed ice-thickness solution, we use a value of 5 times the mean ice thickness^53,56,57^. In order to solve for this without prior knowledge of ice thickness, we iterate between ice-thickness and coupling-length calculations 5 times. In tests that we ran, three iterations were always sufficient for convergence, and applying five iterations permits a factor of safety between these tests and the present application. For the Gantayat *et al*.^32^ basin-divided and whole glacier approaches^32^, the coupling-length is accounted for by the elevation-band averaging.

#### 3b. Basal-shear-stress-based approaches

We may rewrite Eq. 3 to produce an alternative expression relating ice-thickness directly to basal shear stress:

6 $$H=\frac{{\tau }_{b}}{f{\rho }_{i}g{\rm{\sin }}(\alpha )}.$$

This equation forms the basis for the ‘Glacier Bed Topography’ (GlabTop) approach to calculating glacier thickness^31,34,35^. An empirical relationship between glacier-surface elevation range Δ*z*_*i*_ and basal shear stress *τ*_*b*_ is then used to compute ice thickness:

7 $${\tau }_{b}=\left\{\begin{array}{ll}0.5+159.8\Delta {z}_{i}-43.5{(\Delta {z}_{i})}^{2} & {\rm{i}}{\rm{f}}\;\Delta {z}_{i}\le \;1.6\\ 150 & {\rm{i}}{\rm{f}}\;\Delta {z}_{i} > \;1.6\end{array}\right.$$

with *τ*_*b*_ measured in kPa and Δ*z*_*i*_ in km^28,34^. Similarly to the Gantayat *et al*.^32^ basin-divided and whole glacier approaches^32^, we implement this methods on both whole ice caps (GT2 w model) and individual ice-drainage basins (GT2 b model), and iterate between ice thickness and coupling length to calculate the final ice-thickness value.

#### 3c. Mass-conservation-based approach

The conservation-of-mass approach^29^, which has been applied globally^30,35^, may be written as:

8 $$\frac{\partial h}{\partial t}=\mathop{b}\limits^{\cdot }-\nabla \cdot \overrightarrow{q},$$

with [∂*h*/∂*t*] being the change in glacier surface elevation through time, $\mathop{b}\limits^{\cdot }$ being the glacier surface mass balance rate, and $\nabla \cdot \overrightarrow{q}$ being the ice-flux divergence in the horizontal plane^29^. Integrating Eq. 8 over the entire glacier domain Ω gives:

9 $${\int }_{\Omega }\frac{\partial h}{\partial t}{\rm{d}}\Omega ={\int }_{\Omega }\mathop{b}\limits^{.}{\rm{d}}\Omega $$

with the glacier-wide ice-flux divergence being zero. We use the apparent mass balance $\widetilde{b}$^29,30^, equal to the glacier surface mass balance rate $\mathop{b}\limits^{\cdot }$ minus the glacier surface elevation change rate ∂*h*/∂*t* such that:

10 $${\int }_{\Omega }\widetilde{b}{\rm{d}}\Omega =0.$$

The apparent mass balance of each grid cell ${\widetilde{b}}_{i}$ is calculated according to:

11 $${\widetilde{b}}_{i}=\left\{\begin{array}{ll}({z}_{i}-{z}_{0})\cdot {\left[\frac{{\rm{d}}\widetilde{b}}{{\rm{d}}z}\right]}_{{\rm{abl}}} & {\rm{i}}{\rm{f}}\;{z}_{i}\le {z}_{0}\\ ({z}_{i}-{z}_{0})\cdot {\left[\frac{{\rm{d}}\widetilde{b}}{{\rm{d}}z}\right]}_{{\rm{acc}}} & {\rm{i}}{\rm{f}}\;{z}_{i} > {z}_{0}\end{array}\right.$$

with ${\left[{\rm{d}}\widetilde{b}/{\rm{d}}z\right]}_{{\rm{abl}}}$ and ${\left[{\rm{d}}\widetilde{b}/{\rm{d}}z\right]}_{{\rm{acc}}}$ being the vertical mass-balance gradients for the ablation area and the accumulation areas respectively^29,30,35^. *z*_*i*_ represents the elevation of the cell and *z*_0_ represents the elevation of the apparent equilibrium line altitude (ELA), calculated by solving Eq. 10 for *z*_0_ (see Eq. 11) such that the glacier-integrated apparent mass balance is zero. The glacier-width-normalized mean specific ice flux, ${\bar{q}}_{i}$, is then calculated by integrating all upstream apparent mass balance measurements ($\widetilde{b}$) and dividing by the local glacier width^30^. The ice thickness, *H*, is then calculated as:

12 $$H={\left(\frac{n+2}{2{\left(f{\rho }_{i}g\right)}^{n}{A}_{c}^{* }\exp \left(\frac{{Q}_{c}}{R}\left[\frac{1}{T}-\frac{1}{{T}^{* }}\right]\right)}\right)}^{1/(n+2)}{\left(\frac{{\bar{q}}_{i}(1-\beta )}{{\rm{\sin }}{(\alpha )}^{n}}\right)}^{1/(n+2)}.$$

We use the same iteration between ice-thickness and coupling-length as described in the previous methods. In order to ensure a finite and physically meaningful glacier width measurement, we do not use this method on whole ice caps, and only apply it to individual ice-drainage basins (H & F model).

#### Step 3d. Assessment of uncertainties

We evaluate the uncertainty in ice thickness using the Monte Carlo method, considering parameter uncertainties in Eqs. 5, 6 and 12. We conduct *N* = 1000 runs for each method, randomly sampling from the probability distribution of each input parameter (Table 1).

**Table 1:** Parameter probability distributions used in the ice-thickness inversion.

Parameter	Distribution	Reference(s)
*T* (K)	Uniform (268 to 272)	Clapperton, 1990
		Witte, 1995
		Kaser and Osmaston, 2002
		Schotterer *et al*.^38^
		Saberi *et al*.^12^
*n*	Constant (3)	Nye, 1953
		Cuffey and Paterson, 2010
		Qi *et al*.^61^
*f*	Uniform (0.8 to 1)	Haeberli *et al*.^28^
		Cuffey and Paterson, 2010
		Gantayat *et al*.^32^
*β*	Uniform (0 to 0.4)	Huss and Farinotti 2012
		Cuffey and Paterson, 2010
*ρ*_*i*_ (kg/m^3^)	Uniform (830 to 917)	Kaser and Osmaston, 2002
		Thouret *et al*.^40^
		Cuffey and Paterson, 2010
		Tamayo and Arias, 2010
$\frac{d\widetilde{b}}{d{z}_{abl}}$ (a^−1^)	Uniform (0.008 to 0.01)	Farinotti *et al*.^29^
		Huss and Farinotti 2012
		Farinotti *et al*.^35^
$\frac{d\widetilde{b}}{d{z}_{acc}}$ (a^−1^)	Uniform (0.004 to 0.006)	Farinotti *et al*.^29^
		Huss and Farinotti 2012
		Farinotti *et al*.^35^
*g* (m/s^2^)	Constant (9.79)	
*u* (m/yr)	From feature tracking	From stable bedrock statistics

Terms in Eq. 6 and the left-hand parentheses on the the right-hand side of Eqs. 5 and 12 describe ice rheology and physical parameters. To define *A*_*c*_, we vary temperature uniformly between 268 and 272 K based on local temperature data^12,23,38,58,59^. We keep Glen’s flow exponent, *n*, constant at 3^53,60,61^. We vary the shape factor uniformly between 0.8 and 1: this represents a compromise between low lateral drag near the ice cap summits (while acknowledging the presence of nunataks) and higher later drag in the outlet valley glaciers^28,32,53^. We vary ice density uniformly between 743 and 917 kg/m^3^^23,40,53,62^, which is consistent with available ice-density profiles showing a mean ice density of around 830 kg/m^3^ ^23,40^. No estimate of sliding velocity currently exists for any glacier in the study area, so we vary the basal drag correction *β* within a range of 0 to 0.4, corresponding to between 0 and 40% basal sliding^23,30,53^.

For velocity-based inversions, the mean velocity for each point is derived from the process described above in Step 2, and the standard deviation is calculated from standard deviation of measured velocities over non-glaciated terrain. Equation 5 is then solved at each grid cell in the velocity matrix *N* times, and we extract the mean and standard-deviation ice thicknesses at each grid cell from this single-model ensemble. We repeat the same process for basal-shear-stress-based approaches using Eq. 6 and for the mass-conservation-based approach using Eq. 12, and export all mean and standard deviation maps as geotiffs.

We subdivide these ice masses by the river catchments into which they drain, and convert each into its water-equivalent volume (using a mean ice density of 873.5 kg/m^3^, and an ice density range of 830–917 kg/m^3^) to assess the spatially variable significance of these glaciers in regulating discharge^23,40,62^. We use TopoToolbox^55^ to extract a drainage network and drainage basins from the DEM, cropped to a buffered ice-mask. We define the pour points for basin delineation as the boundary of the domain, and repeat the operations for non-rectangular DEMs extending 1 km, 5 km, and 20 km beyond each ice mask. We then sum the ice volume for each drainage basin, discarding basins containing no glaciers.

#### Step 3e. Ensemble mean ice-thicknesses and ice volumes

We calculate six suites of 1000 ice-thickness maps for each of the six methods described above (6000 ice-thickness maps in total):

1. A new, fully distributed two-dimensional ice velocity inversion (VWDV model).
2. An ice-cap-wide ice velocity inversion, with elevation-band averaged surface slope and flow speed (G14 w model)^32^.
3. An velocity inversion, with elevation-band averaged surface slope and flow speed calculated for individual glacier basins (G14 b model)^32,55^.
4. A basal-shear-stress-based approach, with an elevation range calculated for entire ice caps (GT2 w model)^28,31,33–35^.
5. A basal-shear-stress-based approach, with an elevation range calculated for individual glacier basins (GT2 b model)^28,31,33–35,55^.
6. A conservation-of-mass-based approach, with an apparent mass balance calculated for individual glacier basins (H & F model)^29,30,35^.

For each method, we calculate the mean and standard deviation from all Monte Carlo runs. We then calculate a multi-model ensemble mean ice-thickness map of each glacier as the average of the mean ice-thickness maps generated using these six methods. We provide all six individual mean ice-thickness maps alongside the multi-model ensemble mean ice-thickness map. We compare the ice-thickness maps calculated with each of our methods to two prior global compilations, F19^35^ and M22^37^, which we do not include in our multi-model ensemble mean ice-thickness calculation. The accuracy and precision of each map is further discussed in the technical validation section.

For each single-method ice-thickness map and for the multi-model ensemble mean ice-thickness map, we then calculate ice volumes as a simple area-weighted sum:

13 $${V}_{i}=\mathop{\sum }\limits_{j=1}^{{n}_{j}}\mathop{\sum }\limits_{k=1}^{{n}_{k}}\overline{{H}_{jk}}\Delta x\Delta y,$$

where $\overline{{H}_{jk}}$ is either the Monte-Carlo-derived mean ice thickness at each cell or the mean of the six such means, and *dx* and *dy* are the grid resolution along each axis, which in our study remains uniform, but which for the sake of generality we include within the summation.

Similarly, we calculate the standard deviation of the ice volume as an area-weighted sum of the individual standard deviations of ice thickness ($\overline{{\sigma }_{jk}}$) at each grid cell:

14 $${\sigma }_{i}=\mathop{\sum }\limits_{j=1}^{{n}_{j}}\mathop{\sum }\limits_{k=1}^{{n}_{k}}\overline{{\sigma }_{jk}}\Delta x\Delta y.$$

## Data Records

We compile a database of ice velocity, thickness, and volume for every glacier in the Northern Andes calculated using the six methods described above^43^. All ice-thickness maps include an additional assessment of ice-thickness uncertainty.

### Ice velocity

**Fig. 2:**
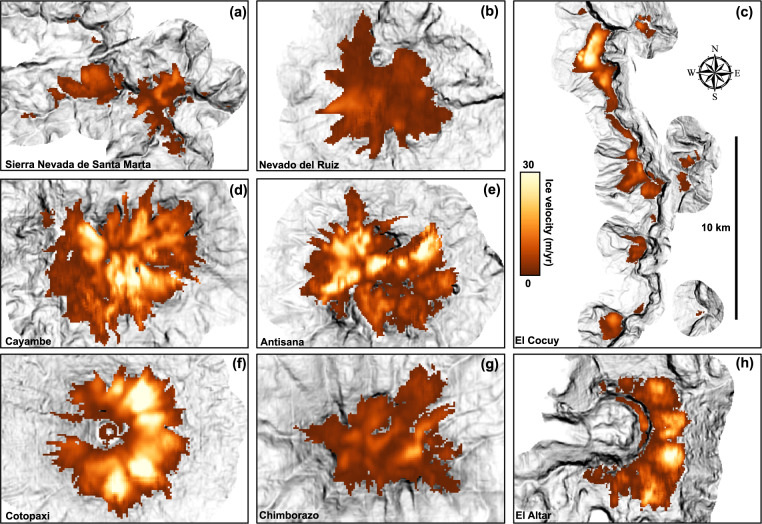
Mean ice velocities of select glaciers in the Northern Andes.(**a**–**c**) Colombian glaciers. (**d**–**h**) Ecuadorian glaciers.

Figure 2 shows mean ice-surface velocities for selected glaciers in the Northern Andes, which range from 0 to 50 metres per year. The fastest flowing ice is located on the western flank of the Cotopaxi ice cap. Apparent velocities over non-glaciated regions, representing the local noise level, are less than 2 meters per year in all cases. This low background noise level enables us to detect motion on even very small and slow moving glaciers. We do not detect flow above background noise on two glaciers, Illiniza Sur (Ecuador) and La Corona (Venezuela), thereby preventing us from calculating meaningful ice-thickness maps and indicating a strong likelihood that only permanent snowfields remain at these locations^63,64^. Due to the small size of these glaciers (<0.1 km^2^), they are of negligible importance to overall ice-volume calculations.

### Ice-thickness and volume

**Fig. 3:**
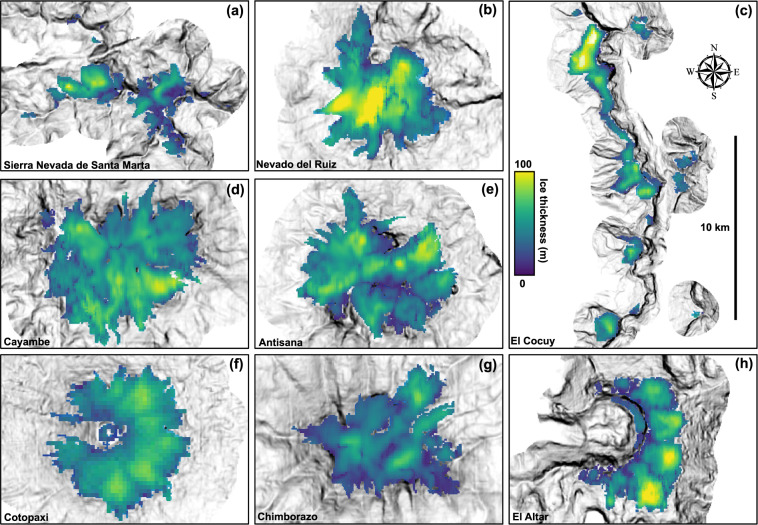
Mean ice-thicknesses of select glaciers in the Northern Andes calculated using the new, fully distributed, velocity-based inversion (VWDV model). (**a**–**c**) Colombian glaciers. (**d**–**h**) Ecuadorian glaciers.

**Fig. 4:**
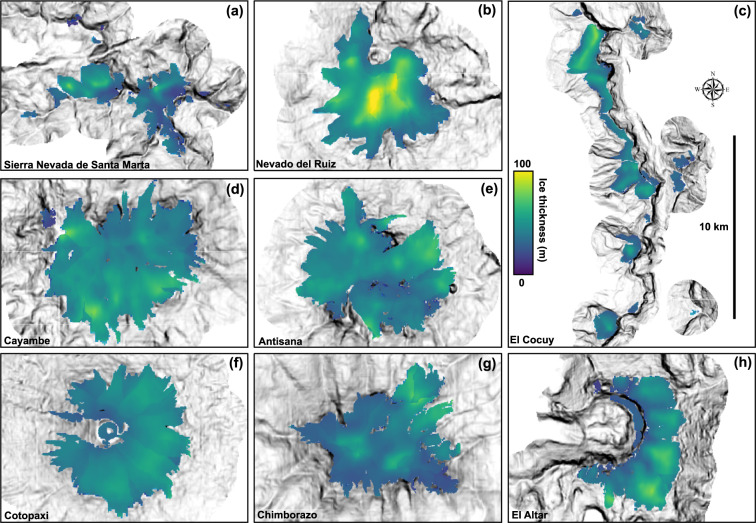
Multi-model ensemble mean mean ice-thicknesses of select glaciers in the Northern Andes, averaging all six methods we use. (**a**–**c**) Colombian glaciers. (**d**–**h**) Ecuadorian glaciers.

Figures 3 and 4 show mean ice-thicknesses maps for the same selected glaciers calculated using the new, fully-distributed velocity-based inversion and the mutli-model ensemble mean respectively. Ice volumes calculated using all six individual methods, multi-model ensemble mean ice volumes, and previous best ice volume estimates^35,37^ are provided in Table 2. Nevado del Ruiz has the highest maximum and average ice-thickness. The ice cap on Volcán Cayambe has the greatest ice volume for any individual ice cap, with estimates ranging from 0.52 ± 0.05 km^3^ (basal-shear-stress-based basin-divided approach^31,34^) to 0.74 ± 0.06 km^3^ (conservation-of-mass-based method^29,30^). Three out of six methods show a greater total volume in the El Cocuy region, although this is spread across multiple distinct ice bodies.

**Table 2:** Area-integrated ice volumes and thicknesses.

Glacier	Volumes (10^7^ m^3^)								
Colombia	Comp	H F	VWDV	GT2 b	GT2 w	G14 b	G14 w	F19	M22
S. N. de Santa Marta	27 ± 6	33 ± 2	29 ± 3	22 ± 2	22 ± 2	29 ± 3	29 ± 3	58	None
El Cocuy	64 ± 19	69 ± 6	75 ± 7	44 ± 4	43 ± 3	76 ± 6	77 ± 6	80	None
Nevado del Ruiz	45 ± 12	47 ± 4	52 ± 6	29 ± 2	40 ± 3	51 ± 5	50 ± 5	119	None
Santa Isabel	3 ± 1.5	3 ± 0.3	4 ± 0.6	1 ± 0.1	1 ± 0.1	4 ± 0.4	4 ± 0.4	18	None
Nevado de Tolima	2 ± 0.6	2 ± 0.2	2 ± 0.3	0.8 ± 0.1	1 ± 0.1	2 ± 0.2	2 ± 0.2	3	None
Nevado del Huila	28 ± 7	31 ± 3	30 ± 3	19 ± 2	22 ± 2	31 ± 3	32 ± 3	53	37 ± 30
All	168 ± 24	184 ± 7	192 ± 10	116 ± 5	128 ± 5	193 ± 9	194 ± 9	330	Incomplete
**Ecuador**	**Comp**	**H F**	**VWDV**	**GT2 b**	**GT2 w**	**G14 b**	**G14 w**	**F19**	
Cayambe	66 ± 12	74 ± 6	68 ± 7	52 ± 5	59 ± 5	69 ± 6	70 ± 6	99	88 ± 60
Antisana	61 ± 12	69 ± 5	63 ± 6	47 ± 4	55 ± 5	64 ± 5	65 ± 6	86	64 ± 46
Cotopaxi	45 ± 10	48 ± 4	50 ± 65	32 ± 3	38 ± 3	50 ± 4	52 ± 5	69	45 ± 36
Chimborazo	34 ± 7	42 ± 3	32 ± 4	28 ± 2	36 ± 3	33 ± 3	33 ± 3	83	None
El Altar	44 ± 12	48 ± 4	50 ± 6	30 ± 3	34 ± 3	51 ± 4	52 ± 5	83	78 ± 57
All	249 ± 25	281 ± 10	263 ± 12	190 ± 8	222 ± 9	266 ± 11	272 ± 11	481	Incomplete
**Total**	**Comp**	**H F**	**VWDV**	**GT2 b**	**GT2 w**	**G14 b**	**G14 w**	**F19**	
All	417 ± 35	465 ± 13	456 ± 16	306 ± 9	350 ± 10	459 ± 14	466 ± 14	811	Incomplete

Comp = multi-model ensemble mean ice-thickness; H F = mass-conservation-based approach^29,30^; VWDV = fully distributed velocity-based inversion from this study; GT2 b = basal-shear-stress-based basin-divided approach^31,34^; GT2 w = basal-shear-stress-based whole glacier approach^31,34^; G14 b = Gantayat *et al*.^32^ basin-divided approach^32^; G14 w = Gantayat *et al*.^32^ whole glacier approach^32^; F19 = Farinotti *et al*.^35^ volumes^35^; M22 = Millan *et al*.^37^ volumes^37^.

**Fig. 5:**
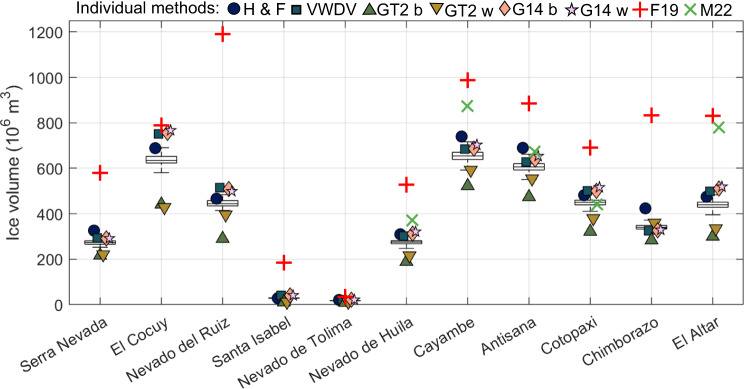
Box plot showing the volume of all glaciers in Ecuador and Colombia. Boxes and error bars represent 1 standard deviation and the 95% confidence interval of the multi-model ensemble mean. Individual symbols represent mean volume estimates for each glacier from the six methods applied in this study and Farinotti *et al*.^35^. H F = mass-conservation-based approach^29,30^; VWDV = fully distributed velocity-based inversion from this study; GT2 b = basal-shear-stress-based basin-divided approach^31,34^; GT2 w = basal-shear-stress-based whole glacier approach^31,34^; G14 b = Gantayat *et al*.^32^ basin-divided approach^32^; G14 w = Gantayat *et al*.^32^ whole glacier approach^32^; F19 = Farinotti *et al*.^35^ volumes^35^; M22 = Millan *et al*.^37^ volumes^37^.

Estimates of total ice volume in Colombia range from 1.16 ± 0.05 km^3^ to 1.94 ± 0.09 km^3^, with the velocity- and conservation-of-mass-based ice-thickness calculations clustering near the upper bound. Estimates of total ice volume in Ecuador range from 1.90 ± 0.08 km^3^ to 2.81 ± 0.10 km^3^. These estimates are around 50% lower than the previous best estimate of 3.30 km^3^ for Colombia and 4.81 km^3^ for Ecuador^35^) (Fig. 5). We cannot calculate regional volumes from the most recent global assessment of glacier thickness^37^, as more than half of the glaciers in this region have no data coverage. The river-catchment-subdivided volumes shows that while some mountains have a near-perfect radial drainage pattern (e.g. Cotopaxi), the majority of the ice is drained eastwards for all Ecuadorian glaciers (Fig. 7)).

## Technical Validation

We evaluate the ice thicknesses and volumes in our database through:

1. Examination of the spatial correlation between individual ice-thickness estimates derived from different methods.
2. Comparison of ice thicknesses calculated in this study to field measurements of ice thickness.
3. Evaluation of the sensitivity of ice-volume measurements to the distribution of input parameters.

### Correlation between ice-volume estimates

**Fig. 6:**
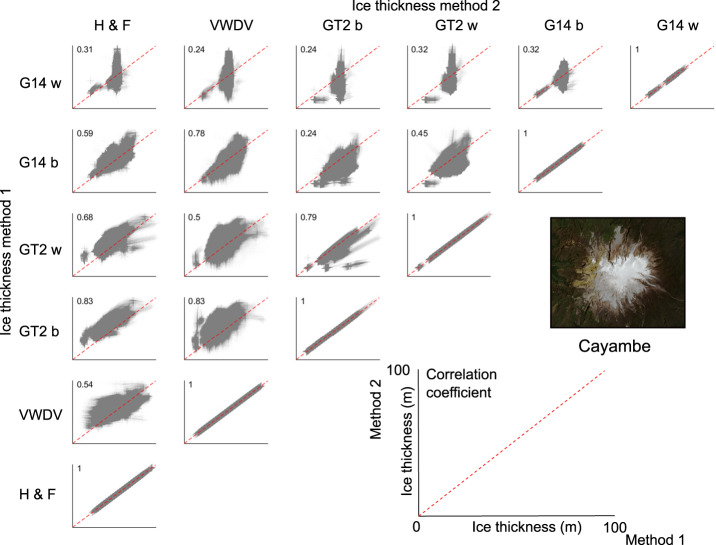
Correlation matrix of the six methods we use to calculate ice thicknesses for Volcán Cayambe. H F = mass-conservation-based approach^29,30^; VWDV = fully distributed velocity-based inversion from this study; GT2 b = basal-shear-stress-based basin-divided approach^31,34^; GT2 w = basal-shear-stress-based whole glacier approach^31,34^; G14 b = Gantayat *et al*.^32^ basin-divided approach^32^; G14 w = Gantayat *et al*.^32^ whole glacier approach^32^.

We evaluate the two-dimensional correlation between the six different methods used to calculate ice thicknesses (Fig. 6). The two dimensional correlation coefficient *C*_2*D*_ is calculated as follows:

15 $${C}_{2D}=\frac{{\sum }_{m}{\sum }_{n}\left({A}_{mn}-\bar{{A}}\right)\left({B}_{mn}-\bar{B}\right)}{\sqrt{({\sum }_{m}{\sum }_{n}{\left({A}_{mn}-\bar{{A}}\right)}^{2})({\sum }_{m}{\sum }_{n}{\left({B}_{mn}-\bar{B}\right)}^{2})}},$$

**Fig. 7:**
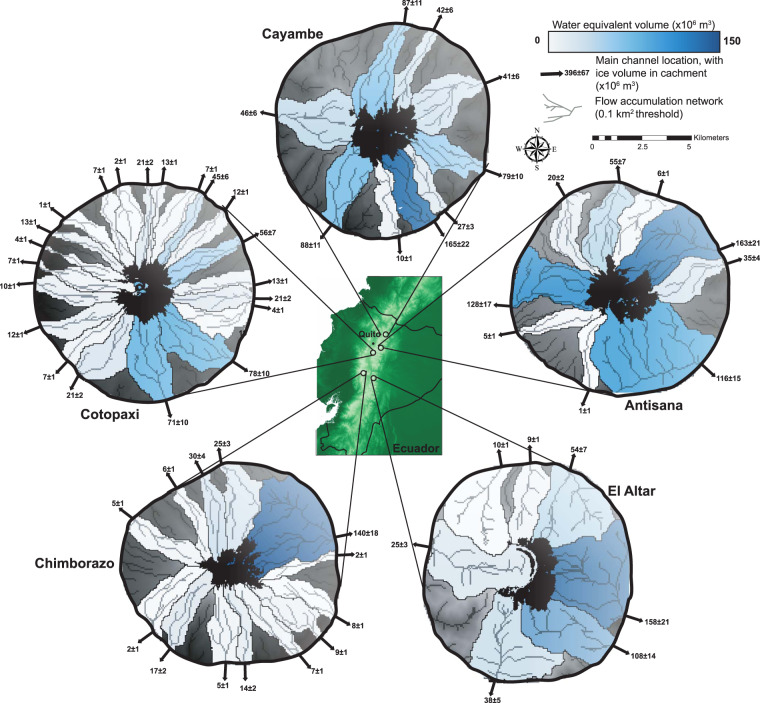
Water-equivalent volumes within different catchments draining the five remaining Ecuadorian glaciers. An ice density range of 830 to 917 kg/m3 (mean of 873.5 kg/m3) was used to convert ice volumes to water-equivalent volumes^23,40,62^.

with A and B being two ice-thickness maps, and *m* and *n* being the x and y direction indices of each ice-thickness measurement. No two ice-thickness maps calculated from different methods have a correlation coefficient greater than 0.9, justifying their usage as distinct estimates of ice-thickness. The mass-conservation-based approach^29,30^ and three velocity-based inversions^32,36^ generally exhibit correlation coefficients greater than 0.5 between themselves. Ice-thickness maps calculated using either the basin-divided or whole-glacier basal-shear-stress-based approaches^31,34^ exhibit the lowest correlation with other methods.

### Comparison to field measurements

We compare our results to data from three field studies: (i) an ice-penetrating-radar-based volume estimate for Nevado del Ruiz^39–41^, (ii) an ice core drilled to bedrock on Volcán Chimborazo^38^, and (iii) multiple ice-penetrating radar lines on Volcán Antisana^42^. Overall, we note that only a small number of on-site field measurements of ice thickness and volume exist in the Northern Andes. In addition, comparisons between our measurements and existing data are complicated by the rapid ice loss that has occurred during the time between the field measurements and the collection of the Sentinel-2 imagery that we use in our thickness inversions.

#### Nevado del Ruiz glacier volumes

**Fig. 8:**
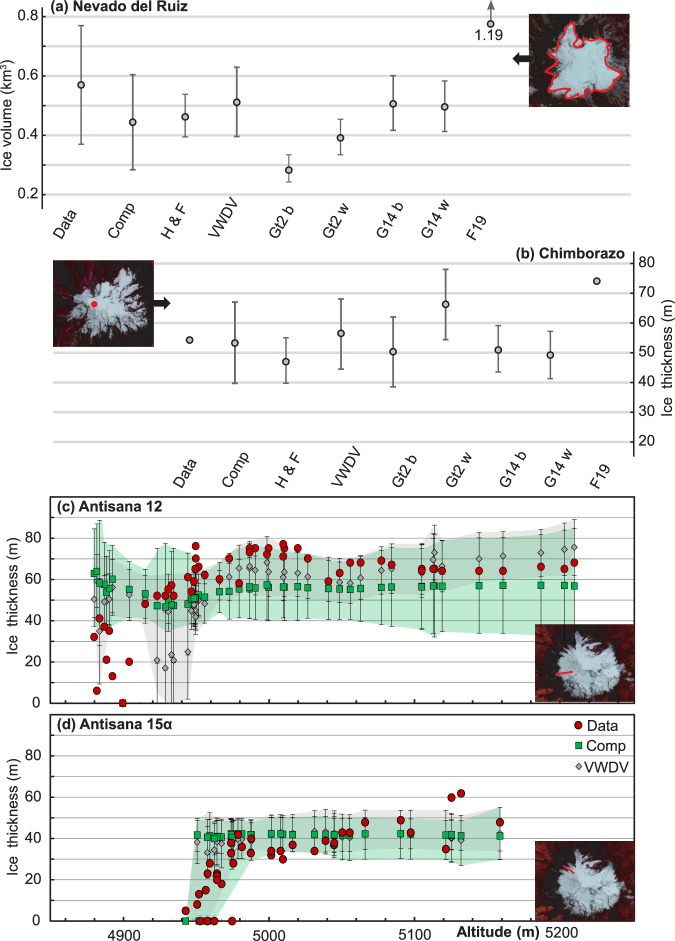
Comparison between ice thicknesses and volumes derived from this study with those from ground-based surveys: (**a**) Nevado del Ruiz^39,40^; (**b**) Chimborazo^38^; (**c**) and (**d**) Antisana^42^. The location of the Chimborazo ice core (Cumbre Veintimilla) and Antisana IPR lines are given by a red dot and red line, respectively. H F = mass-conservation-based approach^29,30^; VWDV = fully distributed velocity-based inversion from this study; GT2 b = basal-shear-stress-based basin-divided approach^31,34^; GT2 w = basal-shear-stress-based whole glacier approach^31,34^; G14 b = Gantayat *et al*.^32^ basin-divided approach^32^; G14 w = Gantayat *et al*.^32^ whole glacier approach^32^; F19 = Farinotti *et al*.^35^ volumes^35^. We do not include data from Millan *et al*.^37^, as they did not calculate the ice thickness or volume for Chimborazo or Nevado del Ruiz^37^.

An ice-penetrating radar (IPR) survey of the Nevado del Ruiz ice cap was conducted in 2000^39–41^. These data provide an ice-cap volume of 0.57 ± 0.20 km^3^, calculated by extrapolating between the existing grid of IPR measurements. We calculate volumes for the Nevado del Ruiz ice cap ranging from 0.29 ±  0.07 km^3^ to 0.52 ± 0.06 km^3^, with a multi-model ensemble mean ice volume of 0.45 ±  0.12 km^3^ (Fig. 8). Total ice-volume loss from the Nevado del Ruiz ice cap between the years 2000 and 2015–2021, calculated from differencing a 20-year timeseries of ASTER DEMs^21^, is 0.15 ± 0.03 km^3^. Subtracting the volume loss from the 2000 IPR survey suggests a current ice volume of 0.42 ± 0.20 km^3^, within uncertainty of our remotely-sensed volume (Fig. 8a). Farinotti *et al*.^35^ calculate a 2000 ice-cap volume of 1.19 km^3^, likely overestimating the ice volume present by a factor of two or more^35,39–41^. Millan *et al*.’s^37^ global compilation does not include any data for Nevado del Ruiz^37^.

#### Chimborazo ice thickness

An ice core was drilled to bedrock on Chimborazo’s second summit, Cumbre Veintimilla, in December 2000^38^. The measured depth to bedrock from this borehole is 54.4 m (see Fig. 8b). We calculate an ice thickness at Cumbre Veintimilla ranging from 47 ± 4 m to 66 ± 6 m, with a multi-model ensemble mean ice thickness of 53 ± 7 m. ASTER-based elevation-change measurements^21^ show an ice-thickness change of +4 ± 19 m over the period 2000–2018 at Cumbre Veintimilla. This would result in a present-day ice-thickness of 58 ± 19 m at this point, matching our remotely-sensed volume (Fig. 8a). Farinotti *et al*.^35^ estimate a 2000 ice-thickness of 73 m for Cumbre Veintimilla, around 20 m thicker than the ice core^35^. Millan *et al*.’s^37^ global compilation does not include any data for Chimborazo^37^.

#### Antisana ice-thickness

IPR surveys were conducted between December 2013 and March 2014 on two outlet glaciers of Volcán Antisana:^42^ 12 and 15 *α*. Located to the NW and W of Antisana’s peak, respectively, they contribute to the water supply of Ecuador’s capital, Quito^5,44^ (Fig. 8c,d). We compare data from our inversions at the same points as the field-based ice-thickness measurements.

For outlet glacier 12, we find a mean ice thickness across all points where IPR was collected ranging from 46 ± 8 m to 60 ± 9 m. Our velocity-based inversion provides a mean ice thickness of 54 ± 16 m, and the multi-model ensemble mean mean ice thickness is 54 ± 16 m at these points. All of these values are within error of the March 2014 mean ice thickness of 58 ± 18 m for all IPR measurements (Fig. 8c). Farinotti *et al*.^35^ and Millan *et al*.^37^ calculate a mean ice-thickness of 75 ± 7 m and 66 ± 38 m respectively for the same points. The two basal-shear-stress-based methods^31,34^, the Gantayat *et al*.^32^ basin-divided approach^32^, and Farinotti *et al*.’s^35^ ice-thickness map^35^ have correlation coefficients of less than 0.05 when compared point-wise with the IPR data, showing no correspondence with the measured spatial pattern of ice thickness. The mass-conservation-based approach^29,30^, our new ice-velocity inversion, and the multi-model ensemble mean ice thickness show positive correlations of 0.74, 0.45 and 0.32 with the IPR data respectively.

For outlet glacier 15 *α*, we find a mean ice thickness across all points where IPR was collected ranging from 29 ± 12 m to 41 ± 17 m. Our new velocity-based inversion provides a mean ice thickness of 34 ± 14 m, and the multi-model ensemble mean ice thickness is 37 ± 14 m at these points. All of these values are within error of the March 2014 mean ice thickness^42^ of 30 ± 16 m for all IPR measurements (Fig. 8d). Farinotti *et al*.^35^ and Millan *et al*.^37^ calculate a mean ice thickness of 30 ± 3 m and 17 ± 14 m respectively for the same points^35,37^. All methods have a positive point-wise correlation coefficient with the IPR-derived ice thicknesses, with the two basal-shear-stress-based approaches having the lowest correlation coefficient (0.37–0.38). The mass-conservation-based approach^29,30^ and our new ice-velocity inversion exhibit the highest correlation coefficients of 0.64 and 0.55 respectively, and the multi-model ensemble mean ice thickness has a correlation coefficient of 0.43.

The comparison presented here shows that our remotely sensed ice thicknesses and volumes are consistent with field-based data. Certain models provide a better match to the data than others, with the multi-model ensemble mean, mass-conservation-based approach^29,30^, and distributed ice-velocity inversion showing the best results. All approaches requiring division of ice caps into individual glacier basins result in artificial step changes in thickness at the boundaries between basins. We therefore recommend that studies requiring the two-dimensional pattern of glacier thickness use the ice-thickness maps produced using our new fully-distributed velocity-based inversion, while studies requiring only the glacier-wide or regional ice volume use either our new fully-distributed velocity-based inversion or the multi-model ensemble mean ice thickness maps^33,65^.

### Sensitivity analysis

**Fig. 9:**
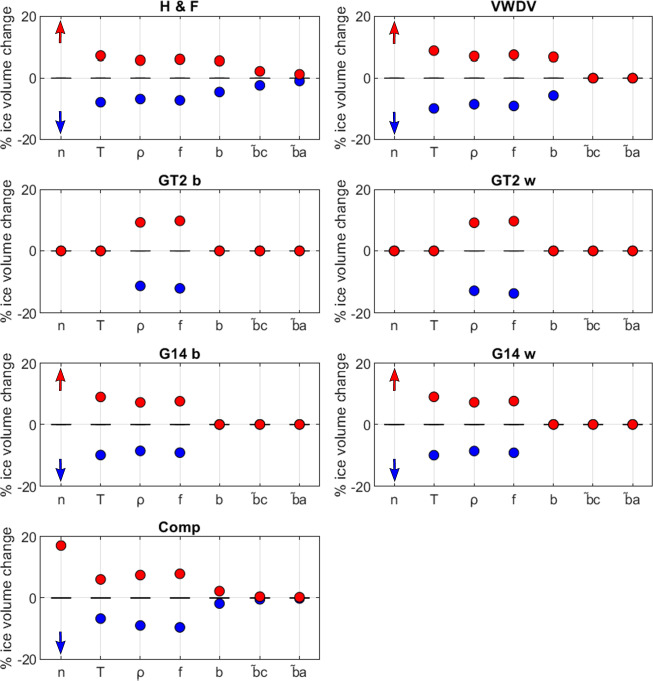
Sensitivity of Volcán Cayambe’s ice volume to the distribution of different parameters. Parameter distributions are given in Table 1. For the purpose of this sensitivity test, we vary the value of Glen’s flow exponent, though in the remainder of our investigation we hold it at a constant value of 3. Comp = multi-model ensemble mean ice-thickness; H F = mass-conservation-based approach^29,30^; VWDV = fully distributed velocity-based inversion from this study; GT2 b = basal-shear-stress-based basin-divided approach^31,34^; GT2 w = basal-shear-stress-based whole glacier approach^31,34^; G14 b = Gantayat *et al*.^32^ basin-divided approach^32^; G14 w = Gantayat *et al*.^32^ whole glacier approach^32^.

To further evaluate potential sources of bias within our ice-thickness dataset, we run a comprehensive sensitivity analysis on all parameters used for the ice-thickness calculation. For each parameter evaluated, we hold all other parameters constant at their mean value, vary the chosen parameter within the range used in this study (Table 1), and evaluate the sensitivity of glacier-integrated ice volume for all parameters. We vary temperature (268 to 272 K), ice density (743 to 917 kg/m^3^), the lateral drag factor (0.8 to 1), the basal sliding correction (0 to 0.4), and the vertical mass-balance gradients for the ablation area and the accumulation areas (0.008 to 0.01 and 0.004 to 0.006 respectively). We also vary the value of Glen’s flow exponent from the constant value of 3 used in this study for investigative purposes, within a range of 2.9 to 3.1. For ease of comparison between different mean glacier volumes, we evaluate the sensitivity ∂*H* as a percentage change:

16 $$\partial H=100\frac{{V}_{i}}{{V}_{i(ref)}}$$

with *V*_*i*_ being the ice volume resulting from a specific parameter combination and *V*_*i*(*ref*)_ being a reference ice volume (calculated with all parameters equal to their mean value). Ice volume is most sensitive to temperature, ice density, and the lateral drag factor (*f*), with sensitivities of 5 to 10% to these parameters in isolation (Fig. 9). Ice density and lateral drag factor have the largest sensitivity for the basal-shear-stress-based approaches^31,34^, as they are the only parameters used by this model. Varying Glen’s flow-law exponent *n* by 0.1 affects final ice volumes by 20% or more, highlighting the importance of this value. Changes in vertical mass-balance gradient only affect the mass-conservation-based approach^29,30^, and have a small effect (<5%). A table providing the results of the sensitivity test for each individual glacier region is available in the data record^43^.

## Usage Notes

All ice-velocity, ice-thickness, ice-thickness-uncertainty, and basin-divided water-equivalent-volume grids may be downloaded from the Zenodo repository^43^. Individual ice-thickness maps are available for each glaciated area using the six individual methods described in this study, alongside the multi-model ensemble mean ice-thickness map. All data are saved in 32-bit floating-point geotiff format. The data are freely available under the Creative Commons Attribution Licence, CC BY 4.0.

## Data Availability

The feature-tracking code used to derive ice velocities, GIV, is available on github and Zenodo^36,66^. All other code, including Google Earth Engine download scripts and the ice-thickness inversion code, is available on zenodo^67^.
